# Distinct 5′ and 3′ coverage biases shape transcriptome interpretation in Nanopore direct RNA versus PCR-cDNA sequencing

**DOI:** 10.1186/s12864-026-13184-x

**Published:** 2026-07-15

**Authors:** Rebecca E. Lane, Eleanor Calcutt, Anandagopal Srinivasan, Vicki Gamble, Udo Oppermann, Jianfeng Sun, Adam P. Cribbs

**Affiliations:** 1 Botnar Research Centre, Nuffield Department of Orthopaedics, Rheumatology and Musculoskeletal Sciences, Research Unit (BRU), National Institute of Health Research Oxford Biomedical, University of Oxford, Oxford, UK; 2 Oxford Genetics Laboratories, Oxford University Hospitals NHS Foundation Trust, Oxford, UK; 3 Oxford Centre for Translational Myeloma Research University of Oxford, Oxford, UK

**Keywords:** RNA-seq, Direct RNA-seq, Oxford Nanopore, Long-read

## Abstract

Long-read RNA sequencing enables isoform-resolved transcriptomics, but library preparation introduces systematic biases that shape biological interpretation. We benchmarked Oxford Nanopore’s two protocols—PCR-cDNA and direct RNA—using SKMM2 myeloma cells stimulated with interleukin-6 (IL-6) and ERCC synthetic spike-ins. Direct RNA produced longer, higher-quality reads and more high-confidence isoforms, but showed pronounced 5′ coverage loss. PCR-cDNA yielded shorter fragments with 3′ underrepresentation, detecting more low-abundance transcripts at reduced confidence. Protocol-specific biases had major consequences: differential expression analysis revealed limited overlap in IL-6–responsive genes, and pathway enrichment was broader in direct RNA. At the isoform level, differential transcript usage was almost entirely protocol-specific, with case studies (e.g. RPL22L1, GRB2, RNF220) illustrating concordance and divergence. ERCC controls confirmed these biases as technical rather than biological. Together, our results show that while both methods provide accurate gene-level quantification, transcript-level conclusions depend critically on protocol choice, highlighting the need for careful selection in long-read transcriptomics.

**Supplementary Information:**

The online version contains supplementary material available at https://doi.org/10.1186/s12864-026-13184-x.

## Introduction

Long-read sequencing technologies have transformed transcriptomic research by enabling direct measurement of full-length RNA molecules and their isoforms [1]. Among these, Oxford Nanopore Technologies (ONT) has developed protocols capable of sequencing either cDNA molecules generated from reverse-transcribed RNA [2, 3], or native RNA molecules themselves [4–8]. These two approaches—PCR-cDNA and direct RNA sequencing—differ markedly in library preparation, sequencing chemistry, throughput, and bias profiles. Understanding these differences is critical for researchers seeking accurate transcriptome characterisation.

The ONT PCR-cDNA protocol benefits from relatively low input requirements, high throughput, and robust detection of low-abundance transcripts. However, the multiple enzymatic steps required—reverse transcription, strand-switching, ligation and PCR amplification—introduce artefacts [9]. These include preferential amplification of shorter molecules, underrepresentation of GC-rich regions, and incomplete reverse transcription leading to truncated reads. Such biases are well documented in short-read cDNA sequencing and remain a challenge for nanopore-based approaches [10, 11].

Direct RNA (dRNA) sequencing eliminates the need for PCR amplification, allowing direct sequencing of native RNA molecules. This provides several advantages: removal of PCR-induced artefacts, improved isoform resolution through long contiguous reads, and the unique ability to detect base modifications such as N6-methyladenosine [12, 13]. However, these benefits come with trade-offs, including higher input requirements, lower throughput, multiplexing restrictions and reduced read accuracy compared to PCR-cDNA.

Benchmarking studies have demonstrated that both protocols provide accurate gene-level quantification [14], but their biases diverge at the transcript and isoform levels [15]. Consequently, biological inferences—particularly regarding alternative splicing, isoform switching, or differential transcript usage—may depend heavily on the chosen protocol.

In this study, we perform a systematic comparison of Oxford Nanopore Technologies (ONT) PCR-cDNA and dRNA sequencing protocols. We first benchmark both methods in the SKMM2 multiple myeloma cell line stimulated with interleukin-6 (IL-6), providing a controlled model to evaluate how protocol-specific biases impact gene expression, isoform detection, and pathway-level interpretation. This analysis reveals areas of concordance—such as gene-level quantification—and key points of divergence, particularly in transcript isoform usage and differential expression outcomes. To validate that these biases arise from technical rather than biological sources, we extended our evaluation using ERCC synthetic spike-in controls. These confirmed differences in sensitivity, coverage uniformity, and quantitative accuracy between the two protocols. Together, our findings establish a technical framework for understanding the strengths and limitations of ONT RNA sequencing strategies and for guiding protocol choice in long-read transcriptomic studies.

## Results

### Protocol-dependent read characteristics and transcript detection in SKMM2 myeloma cells

**Fig. 1:**
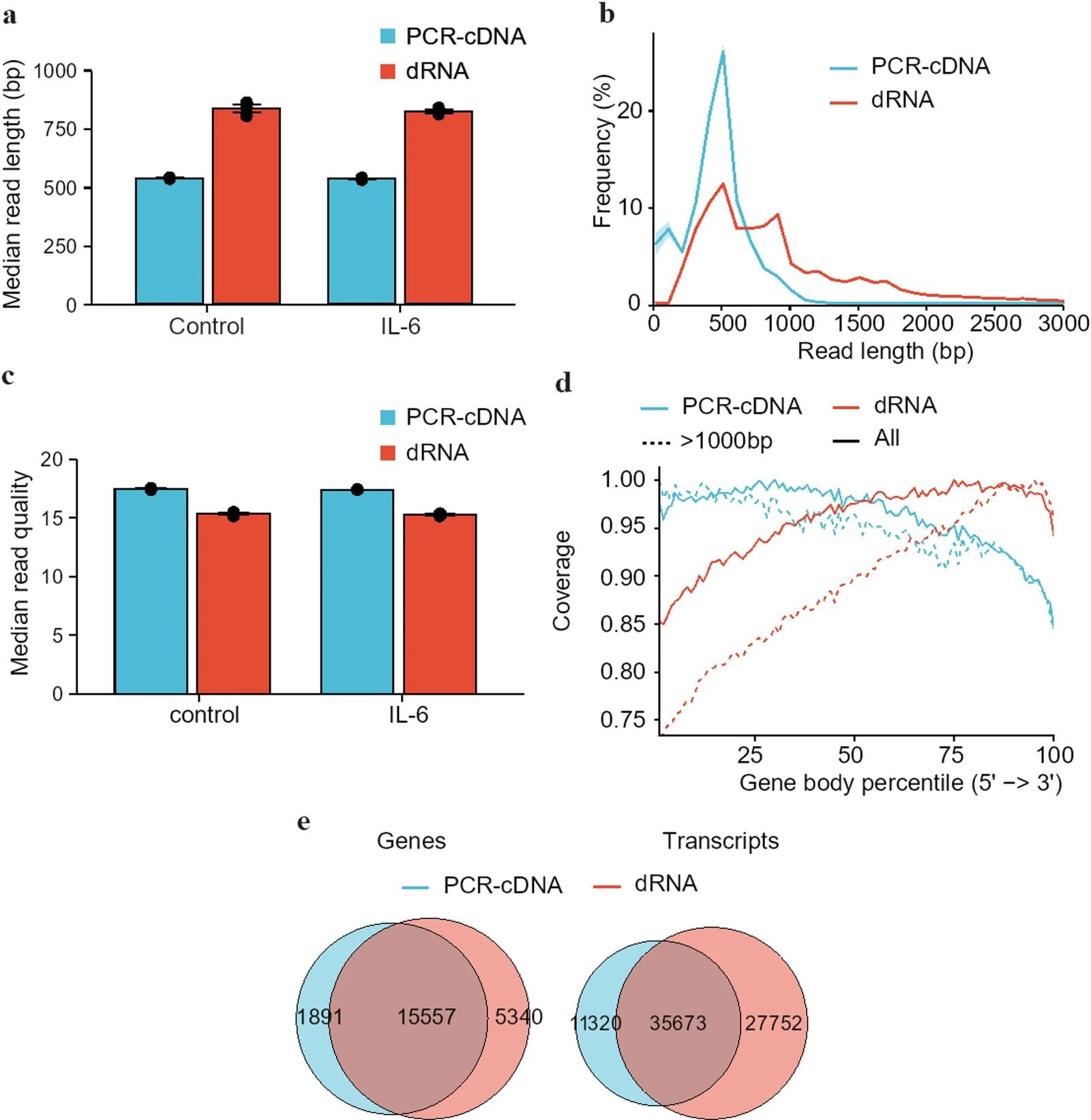
Read characteristics and transcriptome coverage differ between PCR-cDNA and dRNA sequencing. **a** Median read length across biological replicates of SKMM2 myeloma cells cultured with or without IL-6 stimulation. dRNA (red, *N* = 3 per treatment) yielded consistently longer reads than PCR-cDNA (blue, *N* = 3 per treatment). **b** Distribution of median read lengths (*N* = 6 per protocol), showing enrichment of short fragments in PCR-cDNA and broader length representation in dRNA. **c** Median read quality scores by protocol, with dRNA (red, *N* = 3 per treatment) achieving slightly higher values than PCR-cDNA (blue, *N* = 3 per treatment). **d** Mean normalized coverage along transcript bodies, demonstrating protocol-specific biases: PCR-cDNA underestimates coverage towards the 3′ end, while dRNA shows loss of coverage at the 5′ end. **e** Venn diagrams of gene- and transcript-level detection, showing 15,557 genes shared, with PCR-cDNA enriched for unique low-abundance genes and dRNA identifying more isoforms

To assess protocol-specific differences in nanopore transcriptomics, we first compared PCR-cDNA and dRNA sequencing in SKMM2 cells with or without IL-6 stimulation (Fig. 1). dRNA sequencing consistently generated longer reads, with median read lengths of ~ 800 bp compared to ~ 500 bp for PCR-cDNA (Fig. 1a). Read length distributions revealed distinct profiles: PCR-cDNA was enriched for shorter fragments (~ 500 bp peak), whereas dRNA yielded a broader distribution with greater representation of longer molecules (Fig. 1b). These profiles were likely influenced by Immunoglobulin (IG) transcript enrichment, characteristic of plasma cell malignancies (Supplementary Fig. 1) [16, 17]. Specifically, the dRNA peak mode (925 bp) is close to the predicted length of the rearranged SKMM2 IGK transcript (Supplementary Fig. 1c-d). Furthermore, IGK transcripts contributed to 2.8% and 5.7% of total reads in PCR-cDNA and dRNA samples respectively (Supplementary Fig. 2). Read quality scores were slightly higher for PCR-cDNA (median 17–18), compared to dRNA (median 14–15), with no marked influence of IL-6 treatment (Fig. 1c). However, the proportion of full length transcripts captured by dRNA was lower than PCR-cDNA (Supplementary Fig. 3). Thus, the higher dRNA peak may be attributed to the capture of longer transcript fragments.

Coverage profiling across transcripts highlighted characteristic biases of each protocol. PCR-cDNA showed progressive loss of coverage towards the 3′ end of coding sequence, consistent with incomplete reverse transcription and amplification inefficiency. In contrast, dRNA exhibited reduced coverage at the 5′ end, reflecting the 3′–5′ sequencing orientation and RNA degradation (Fig. 1d).

These technical biases translated into differences in transcript detection, despite normalizing for read depth across both protocols. At the gene level, both methods recovered a comparable number of genes, with 15,557 shared and smaller sets uniquely identified by PCR-cDNA (1891) or dRNA (5340) (Fig. 1e). At the transcript level, however, differences were more pronounced: dRNA identified over 63,000 isoforms, whereas PCR-cDNA identified ~ 46,900, with ~ 35,600 shared between protocols. This suggests that dRNA sequencing may be better able to support isoform-level inference, although differences between the sequencing methods suggest biases within each sequencing approach.

### Transcript detection is strongly protocol-dependent

**Fig. 2:**
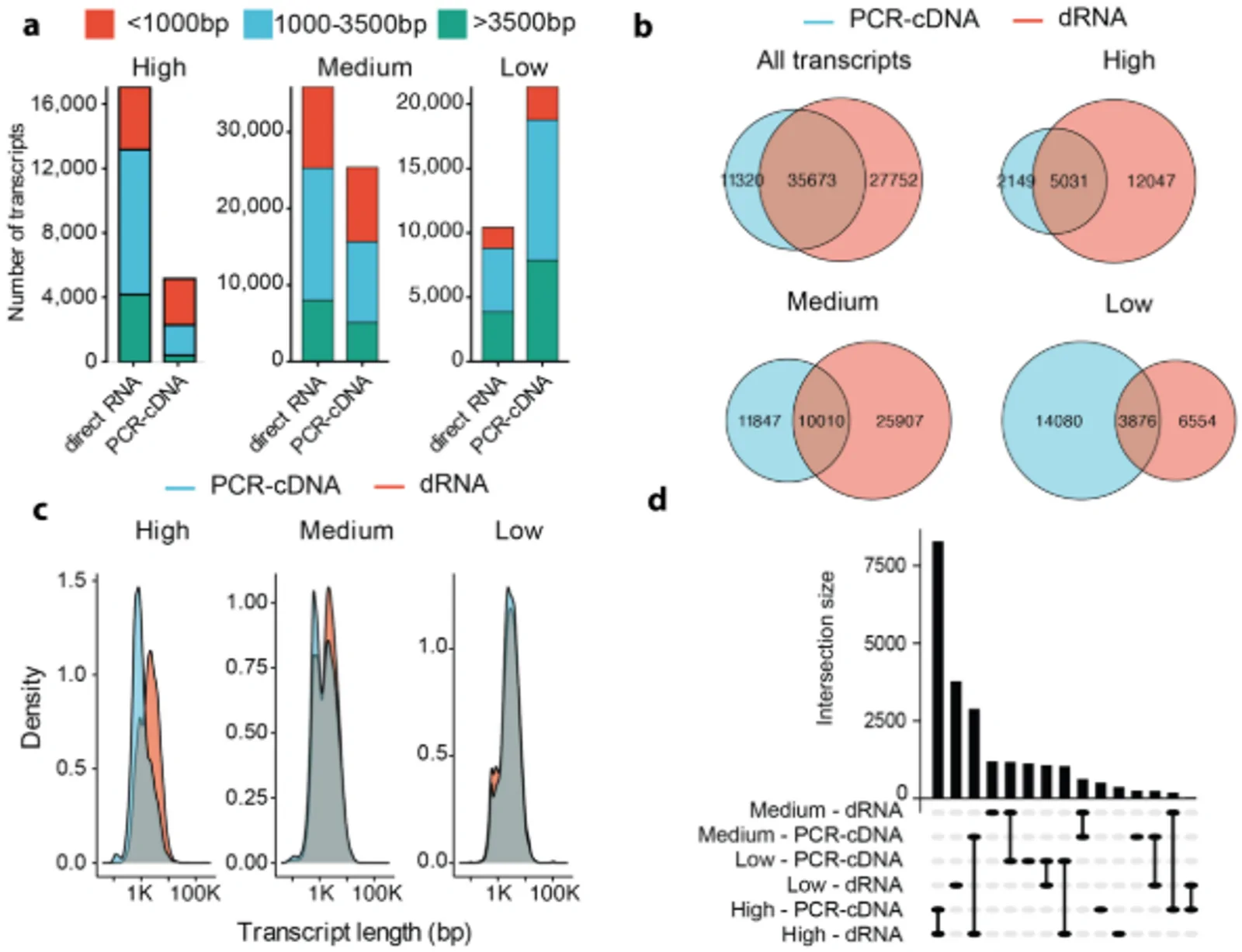
Transcript detection differs by protocol, confidence, and length. **a** Number of transcripts detected by PCR-cDNA and dRNA across high-, medium-, and low-confidence categories in all samples, stratified by transcript length (< 1000 bp, 1000–3500 bp, > 3500 bp). PCR-cDNA is enriched for shorter, low-confidence transcripts, whereas dRNA recovers a greater proportion of longer isoforms. **b** Venn diagrams of transcript detection across protocols. While 37,771 transcripts were shared, dRNA uniquely identified > 25,000 isoforms, particularly at high and medium confidence. PCR-cDNA contributed more unique transcripts at low confidence. **c** Transcript length distributions for high-, medium-, and low-confidence sets. PCR-cDNA libraries are biased towards short isoforms, while dRNA captures a broader size distribution. Overlapping region (grey) between PCR-cDNA and dRNA libraries. **d** Intersection analysis of transcripts across confidence categories and protocols. dRNA shows enhanced recovery of high- and medium-confidence isoforms, while PCR-cDNA contributes disproportionately to low-confidence calls

We next compared the breadth and confidence of transcript detection between protocols. dRNA sequencing identified a greater number of transcripts than PCR-cDNA, with ~ 63,425 versus ~ 46,993 detected, and ~ 35,673 shared between methods (Fig. 2a–b). This broad recovery by dRNA was particularly evident at higher confidence levels. At high confidence, dRNA uniquely detected 12,047 transcripts compared to only 2,149 for PCR-cDNA. At medium confidence, both protocols captured 10,010 shared isoforms, but dRNA contributed more unique transcripts (25,907 vs. 11,847). In contrast, at low confidence PCR-cDNA recovered a much larger fraction of unique transcripts (14,080 vs. 6,554), reflecting increased sensitivity to low-abundance fragments but at reduced assignment certainty.

Transcript length distributions further highlighted protocol-specific biases (Fig. 2c). PCR-cDNA libraries were strongly skewed toward short isoforms (< 1000 bp), particularly at low confidence, consistent with amplification-driven overrepresentation of short molecules. dRNA sequencing, in contrast, recovered a more balanced spectrum of isoform sizes, including robust detection of long transcripts (> 3500 bp).

Intersection analysis confirmed these trends (Fig. 2d). dRNA preferentially contributed high- and medium-confidence isoforms, while PCR-cDNA disproportionately inflated the low-confidence category. Together, these results demonstrate complementary detection properties: PCR-cDNA maximises sensitivity at the expense of confidence and length balance, whereas dRNA sequencing provides broader and more reliable identification of high-confidence, long isoforms.

### Divergent IL-6–induced transcriptional responses across protocols

**Fig. 3:**
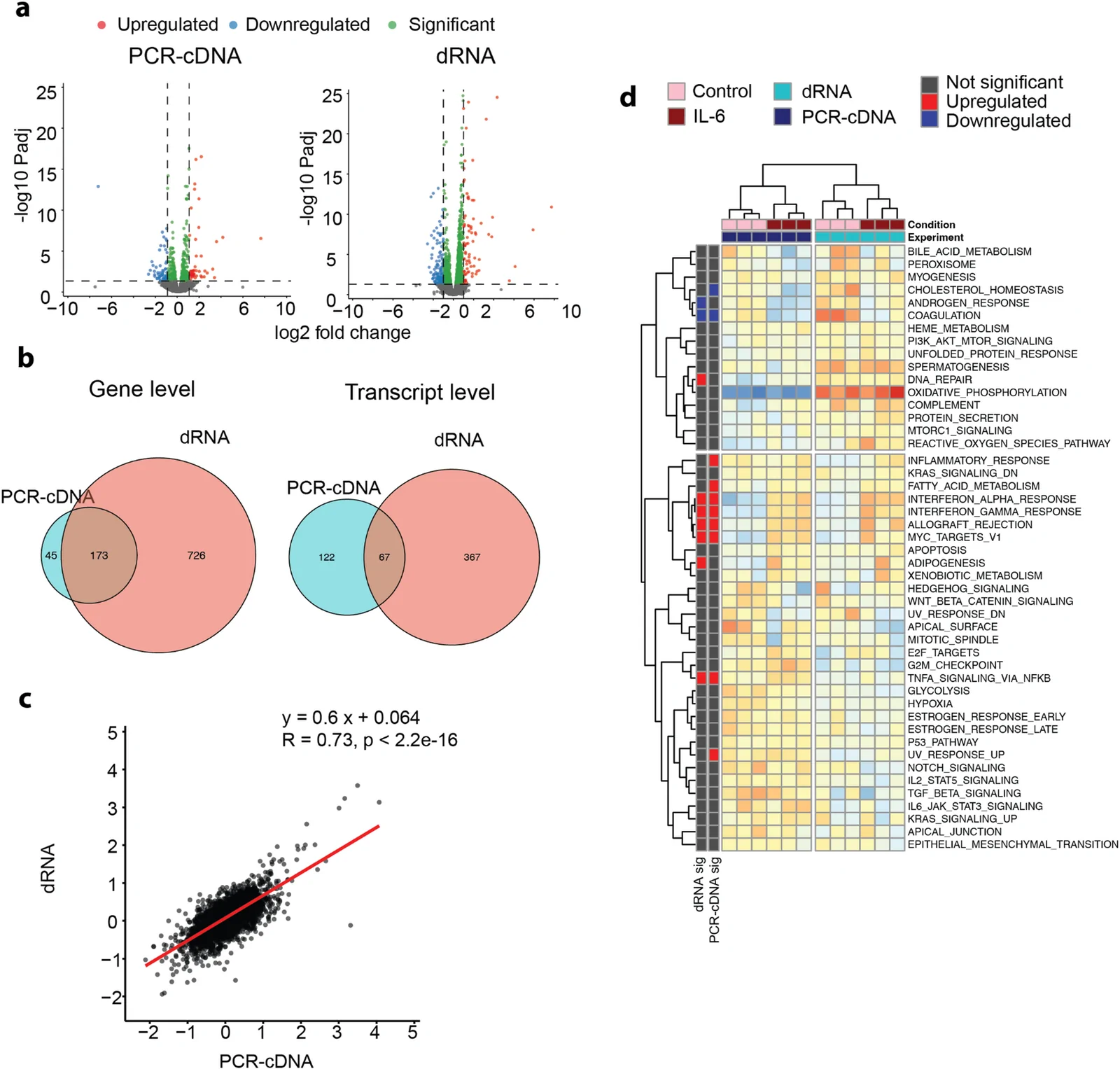
Divergent IL-6–induced transcriptional responses detected by PCR-cDNA and dRNA sequencing. **a** Differential expression analysis of SKMM2 cells stimulated with IL-6. Volcano plots show significantly upregulated (red, log2FC > 1, padj < 0.05) and downregulated (blue, log2FC <-1, padj < 0.05) genes for PCR-cDNA (left, 115 up, 38 down) and dRNA (right, 97 up, 138 down). **b** Venn diagrams at the gene (left) and transcript (right) levels. Overlap between protocols is limited, with only 194 genes and 54 transcripts identified by both, while the majority of differentially expressed features are protocol-specific. **c** Correlation of differential gene expression fold-change estimates between protocols. While overall correlation was moderate (*R* = 0.73), PCR-cDNA compressed fold-change dynamic range relative to dRNA. **d** Pathway-level analysis of differentially expressed genes. Heatmap of hallmark gene set enrichment reveals broader pathway activation with dRNA, including IL-6/JAK–STAT signalling, MYC targets, and inflammatory programmes, whereas PCR-cDNA highlights fewer significantly perturbed pathways

To assess how protocol choice influences biological interpretation, we compared IL-6–stimulated SKMM2 multiple myeloma cells using both PCR-cDNA and dRNA sequencing. Although both approaches detected IL-6–responsive changes, the breadth and concordance of results differed markedly (Fig. 3).

At the differential expression level, PCR-cDNA sequencing identified 218 genes, whereas dRNA sequencing detected substantially more, with 899 genes (Fig. 3a). Overlap between the two approaches was modest: only 173 differentially expressed genes were shared, with the majority uniquely detected by one protocol. This divergence was even more pronounced at the transcript level, where only 67 isoforms were shared, and most differentially expressed transcripts were protocol-specific (Fig. 3b).

Direct comparison of log₂ fold-change values showed only moderate correlation between protocols (*R* = 0.73; Fig. 3c), with PCR-cDNA compressing the dynamic range relative to dRNA. This compression likely contributes to under-detection of isoform-specific responses.

Downstream pathway analysis revealed both shared and protocol-specific patterns of IL-6–responsive signalling (Fig. 3d). While canonical pathways such as IL-6/JAK–STAT signalling, MYC targets, and inflammatory programmes were detected by both approaches, dRNA sequencing generally showed stronger effect sizes and a greater number of statistically significant hallmark gene sets. In contrast, PCR-cDNA identified a subset of these pathways, often with attenuated magnitude of enrichment.

Hierarchical clustering further demonstrated clear separation by sequencing protocol, indicating that protocol-specific technical features influence pathway-level inference. Together, these results suggest that although core IL-6 signalling signatures are reproducible across methods, the breadth and strength of pathway enrichment are shaped by library preparation strategy.

### Protocol-specific detection of differential transcript usage

**Fig. 4:**
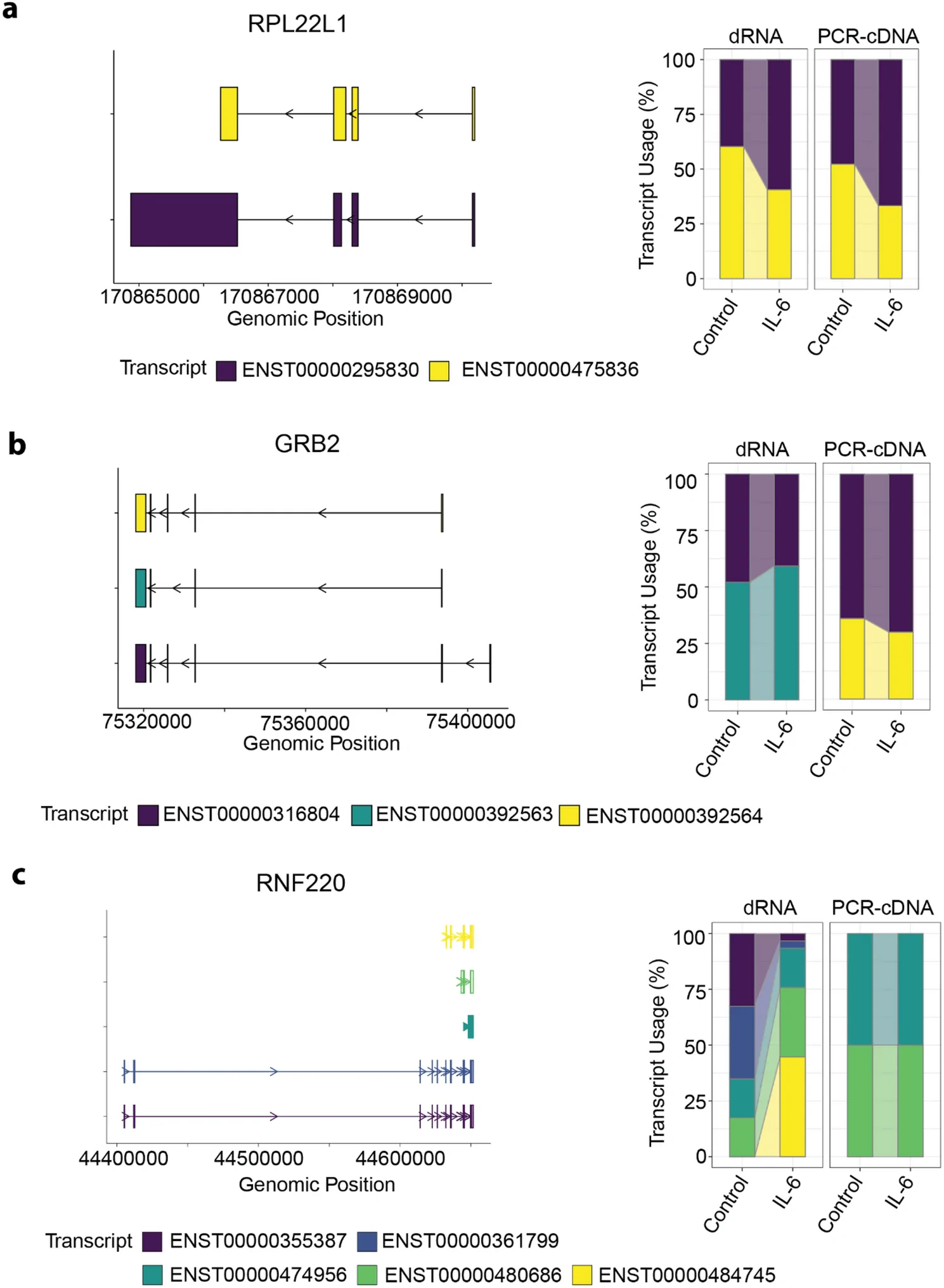
Isoform-specific responses reveal protocol-dependent concordance and divergence. **a** RPL22L1: Two isoforms were detected across conditions. Both PCR-cDNA and dRNA identified shifts in isoform usage upon IL-6 stimulation, with broadly concordant patterns. **b** GRB2: Three isoforms were detected. While both protocols captured IL-6–induced changes, the relative contribution of minor isoforms differed between PCR-cDNA and dRNA. **c** RNF220: Five isoforms were identified. dRNA resolved multiple isoforms with distinct usage patterns, whereas PCR-cDNA predominantly supported two transcripts, underestimating isoform diversity

To further dissect protocol-specific effects, we compared differential transcript usage (DTU) across selected genes (Fig. 4). These examples illustrate both concordance and divergence in isoform-level interpretation between PCR-cDNA and dRNA sequencing.

For RPL22L1, both protocols consistently identified a shift in isoform usage upon IL-6 stimulation, supporting concordant detection of DTU (Fig. 4a). This indicates that for certain highly expressed genes with relatively simple isoform structures, both methods provide reproducible isoform-level inferences.

By contrast, GRB2 showed a striking divergence: PCR-cDNA suggested a switch from ENST00000392564.5 to ENST00000316804.10, whereas dRNA sequencing supported a switch from ENST00000316804.10 to ENST00000392563.5 with limited evidence for switching (Fig. 4b). This discrepancy may arise from PCR amplification bias against longer isoforms.

A different pattern was observed for RNF220, where dRNA sequencing uniquely identified IL-6–induced changes in isoform proportions, whereas PCR-cDNA showed little evidence of DTU (Fig. 4c). This reflects dRNA’s greater capacity to capture long and complex isoforms with higher confidence.

Taken together, these case studies demonstrate that while gene- and pathway-level signals are broadly robust, transcript-level inferences are strongly shaped by the choice of sequencing protocol. The minimal overlap in DTU calls underscores the need for caution when drawing isoform-specific biological conclusions from single-protocol datasets.

### Novel transcript discovery reveals further protocol-specific divergence

**Fig. 5:**
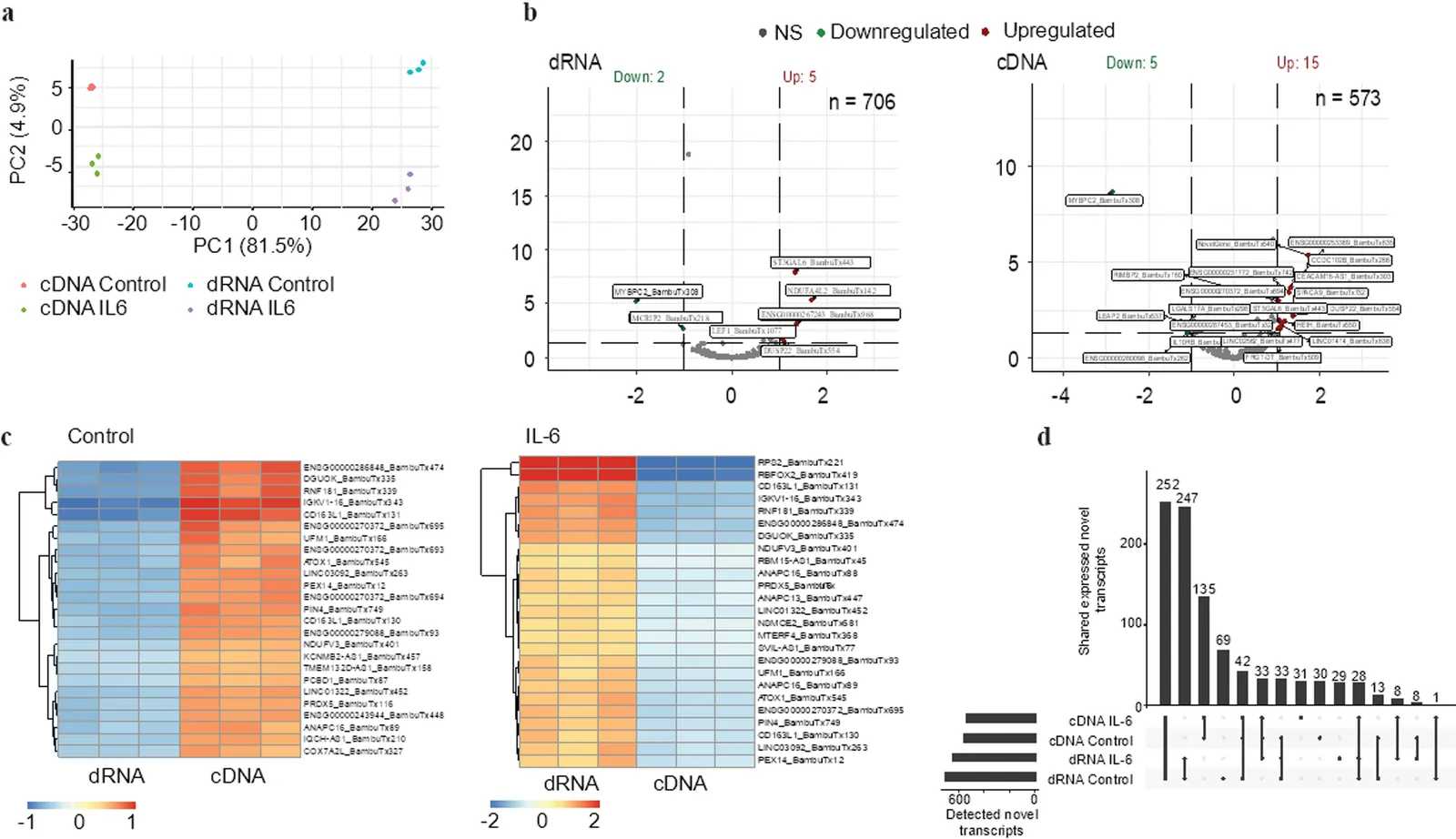
Principal component analysis, differential expression, and detection of novel transcripts across protocols. **a** Principal component analysis (PCA) of SKMM2 cells sequenced by PCR-cDNA or dRNA protocols, with or without IL-6 stimulation, based on expression of novel transcripts. Clear separation by protocol and treatment indicates protocol-specific bias as well as biological response. **b** Differential expression analysis of novel transcripts detected by bambu. Volcano plots show significant upregulated (red, log2FC ≥ 1, padj < 0.05) and downregulated (green, log2FC ≤-1, padj < 0.05) transcripts after IL-6 treatment by PCR-cDNA or dRNA protocols, with labelled examples of significant candidates. **c** Heatmaps of the top differentially expressed novel transcripts of control or IL6 SKMM2 samples by PCR-cDNA or dRNA protocols, illustrating distinct expression patterns across protocols and treatment conditions. **d** Intersection analysis of novel transcript detection across conditions. Bar plots indicate the number of novel transcripts detected per protocol and treatment, while the Upset plot shows shared and unique subsets. dRNA sequencing identified a larger set of novel isoforms compared with PCR-cDNA, with limited overlap between protocols

We next examined the ability of PCR-cDNA and dRNA sequencing to detect and quantify novel isoforms (Fig. 5). Principal component analysis (PCA) showed clear separation by both protocol and treatment, indicating that technical biases and IL-6 stimulation both contribute to the observed variance (Fig. 5a). Differential expression analysis of novel transcripts identified by bambu revealed 20 candidates for PCR-cDNA and 7 candidates for dRNA, only 3 of which showed overlap between protocols (Fig. 5b). Heatmap clustering highlighted distinct expression patterns between dRNA and PCR-cDNA (Fig. 5c), suggesting novel isoform detection is protocol-dependent. Intersection analysis confirmed this trend: dRNA identified the largest set of unique novel isoforms, while overlap between protocols was limited (Fig. 5d). These results demonstrate that protocol choice not only influences detection of annotated isoforms but also strongly affects the discovery and quantification of novel transcripts.

### Detection of fusion transcripts across protocols

**Fig. 6:**
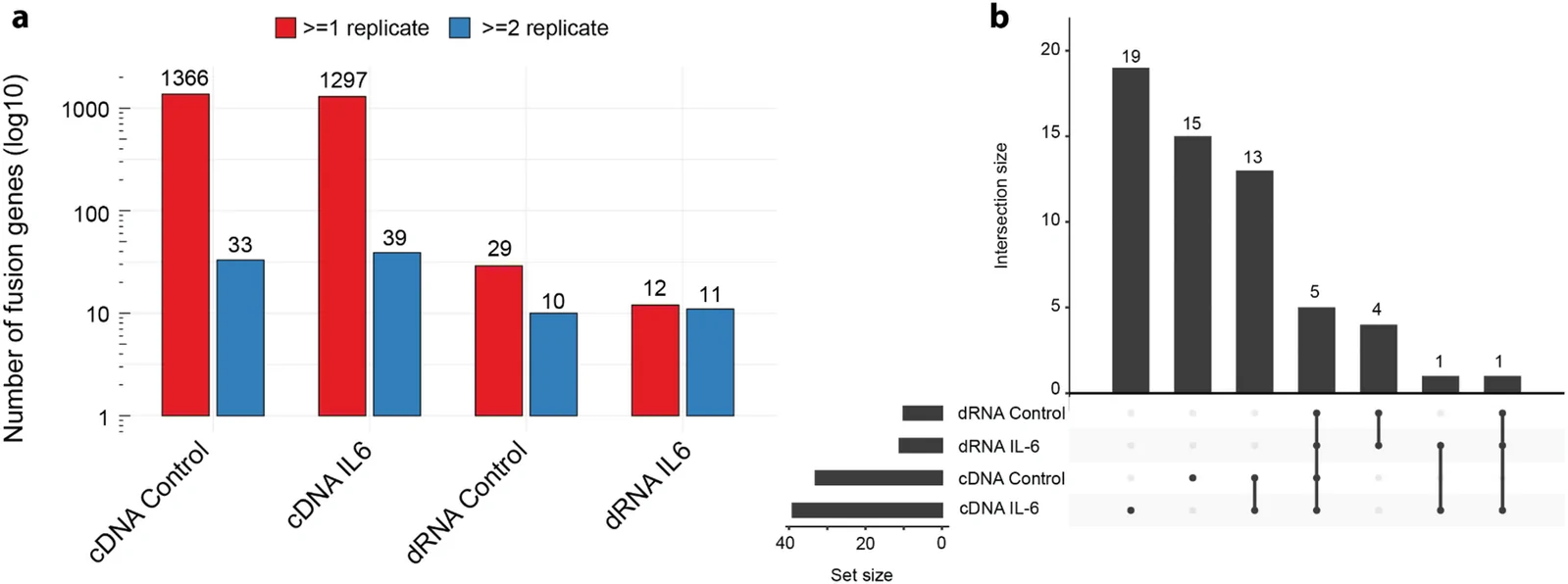
Fusion gene detection differs by protocol. **a** Number of fusion genes detected by PCR-cDNA and dRNA in SKMM2 cells stimulated by IL6 treatment in ≥ 1 or ≥ 2 replicates. **b** Intersection analysis of gene fusions detection across conditions in ≥ 2 replicates. Bar plots indicate the number of fusion genes detected per protocol and treatment, while the Upset plot shows shared and unique subsets. PCR-cDNA sequencing identified a larger set of fusion genes compared with dRNA, with limited overlap between protocols

Long-read RNA sequencing facilitates the accurate detection of fusion genes. We next assessed fusion detection by PCR-cDNA and dRNA long-read sequencing (Fig. 6). Fusion calls were restricted to high-confidence events supported by consensus across five independent pipelines per condition. PCR-cDNA identified over three-fold more candidate fusions than dRNA (Fig. 6a). In control samples, 1,366 fusion candidates were detected by PCR-cDNA in at least one replicate compared to 29 by dRNA. However, requiring detection in ≥ 2 replicates reduced PCR-cDNA calls to 33 (2.4% reproducibility), whereas dRNA retained 10 events (34% reproducibility), indicating substantially greater cross-replicate stability in dRNA. Across both conditions, 58 fusions met reproducibility criteria (Fig. 6b). Of these, 47 were unique to PCR-cDNA, 4 were unique to dRNA, and only 7 were shared between protocols (~ 12% concordance), demonstrating limited inter-protocol agreement.

Importantly, this SKMM2 cell line harbours the canonical t(11;14) translocation and is not known to contain additional recurrent fusions based on prior short-read analyses. In this context of low expected fusion burden, the marked excess of PCR-cDNA fusion calls, particularly those lacking reproducibility, is most consistent with amplification-associated artefacts. By contrast, dRNA sequencing yields fewer candidates but exhibits substantially higher reproducibility.

### Synthetic ERCC benchmarking validates protocol-specific biases

**Fig. 7:**
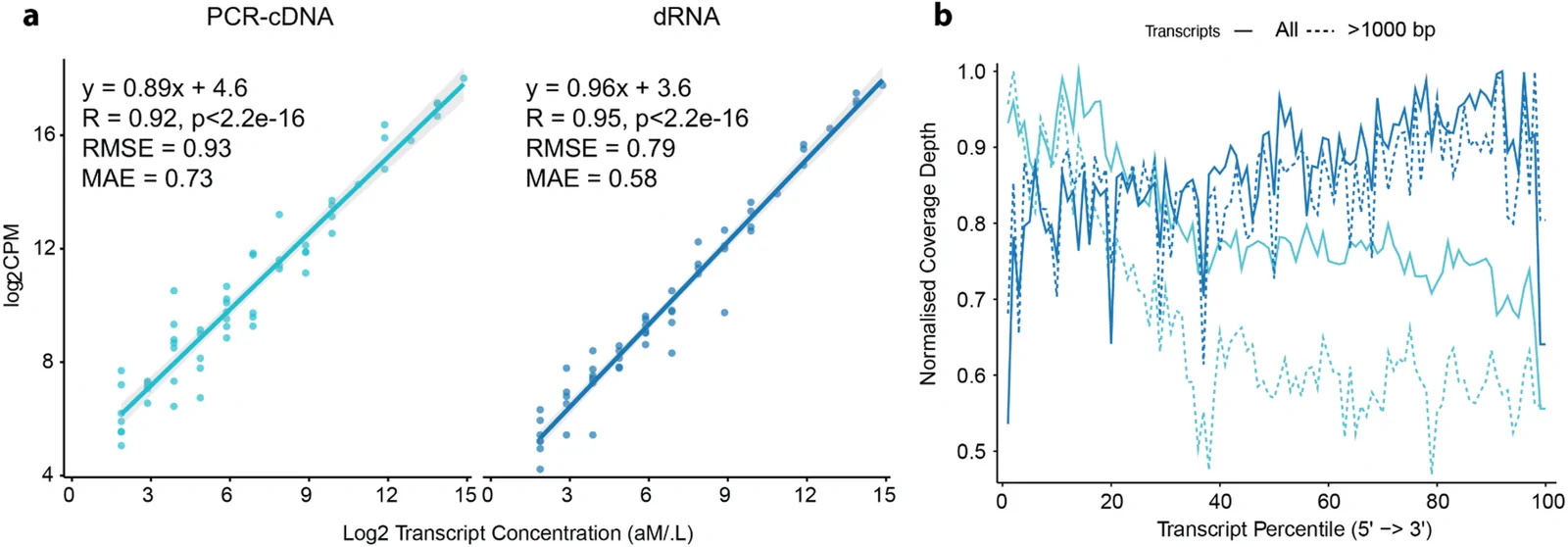
ERCC spike-in controls validate protocol-specific biases. **a** Correlation of measured expression (log₂ CPM) with known ERCC transcript concentrations for PCR-cDNA (left) and dRNA (right). Both protocols achieved strong correlations (*R* = 0.92 for PCR-cDNA, *R* = 0.95 for dRNA), but dRNA showed lower root mean square error (RMSE = 0.79) and mean absolute error (MAE = 0.58) compared with PCR-cDNA (RMSE = 0.93, MAE = 0.73), indicating higher quantitative accuracy. **b** Normalized coverage profiles of ERCC transcripts reveal protocol-specific biases consistent with cellular transcriptomes. PCR-cDNA libraries show underrepresentation towards the 3′ end, whereas dRNA exhibits reduced coverage at the 5′ end. These effects are observed across all spike-ins and are more pronounced in longer transcripts (> 1000 bp)

To disentangle biological variability from protocol artefacts, we sequenced ERCC synthetic spike-in controls using both PCR-cDNA and dRNA protocols (Fig. 7). At the gene level, both protocols produced highly accurate quantification, with transcript abundances strongly correlated with expected concentrations (*R* = 0.97 for PCR-cDNA; *R* = 0.98 for dRNA). Root mean square error (RMSE) and mean absolute error (MAE) values were lower for dRNA (RMSE = 0.68, MAE = 0.47) compared with PCR-cDNA (RMSE = 0.86, MAE = 0.66), confirming a slight higher quantitative accuracy of dRNA (Fig. 7a). ERCCs represent a defined, relatively simple, transcript set with limited structural complexity which may explain why transcript detection is similar between protocols.

Coverage profiles, however, recapitulated the characteristic biases of each protocol (Fig. 7b). PCR-cDNA showed progressive coverage loss at the 3′ ends of spike-ins, consistent with incomplete reverse transcription and PCR artefacts. Conversely, dRNA sequencing displayed reduced coverage at the 5′ ends, reflecting sequencing orientation and RNA degradation. These biases were consistent with those observed in SKMM2 myeloma cells, validating them as intrinsic to the protocols rather than driven by biological features.

Together, the ERCC benchmarking confirms that while both methods provide accurate abundance estimates at the gene level, protocol-specific biases in coverage and isoform detection systematically shape transcriptome interpretation.

## Discussion

This study highlights both the complementary strengths and the limitations of ONT PCR-cDNA and dRNA sequencing when applied to isoform-resolved transcriptomics. Using SKMM2 multiple myeloma cells as a controlled model, we demonstrate that while both protocols capture IL-6–induced transcriptional responses, they diverge substantially at the isoform and transcript level. dRNA sequencing consistently yielded longer reads, more balanced transcript coverage, and higher-confidence isoform assignments, whereas PCR-cDNA provided greater sensitivity for low-abundance transcripts but at the cost of amplification bias and reduced length representation. These protocol-specific biases were independently validated using ERCC synthetic spike-ins, which confirmed that PCR-cDNA libraries tend to enrich for shorter fragments and favour 5′ coverage, while dRNA libraries underrepresent 5′ regions, consistent with RNA degradation.

A further source of technical bias arises during the reverse transcription step of PCR-cDNA library preparation. Incomplete reverse transcription can lead to 3′ truncation of cDNA molecules, which in turn may partially or completely remove the unique molecular identifier (UMI) sequence. As we and others have shown previously [9, 18, 19], this truncation introduces a significant bioinformatic challenge, impeding the accurate identification and collapsing of reads derived from the same RNA molecule. Reads with truncated or unrecognisable UMIs are frequently discarded, resulting in the under-representation of valid long molecules in the final dataset. This not only reduces the accuracy of transcript quantification but also diminishes statistical power for downstream analyses. Such effects are a plausible mechanistic explanation for the weaker identification of differentially expressed genes by the PCR-cDNA method compared to dRNA sequencing.

A notable consequence of these biases is their impact on biological interpretation. At the gene and pathway levels, both protocols recovered canonical IL-6–responsive signatures such as JAK/STAT3 and MYC targets, though dRNA consistently detected broader sets of differentially expressed genes and pathways. At the isoform level, however, overlap was minimal: differential transcript usage (DTU) calls were largely protocol-specific, and novel transcript discovery and candidate fusions diverged between approaches. These findings emphasise that transcript-level biology inferred from long-read sequencing may reflect protocol-specific technical artefacts as much as genuine biology.

Our findings align with and extend two recent benchmarking studies. Pardo-Palacious et al. [15] compared dRNA and cDNA approaches and highlighted challenges in transcript isoform detection, quantification and de novo transcript detection, with dRNA sequencing but also emphasised its lower sensitivity. Similarly, Chen et al. [14] underscored protocol-specific biases in coverage, with dRNA limited by throughput and input requirements. Importantly, this study showed that long-read RNA sequencing facilitates end-to-end reconstruction of fusion transcripts, overcoming limitations of short-read sequencing. Building on this, we demonstrate a dRNA approach provides a higher reproducibility of fusion detection and eliminates amplification-associated artefacts observed with PCR-cDNA libraries. Both studies, like ours, stress that while gene-level quantification is broadly concordant between protocols, isoform-level interpretation is highly protocol-dependent. Crucially, our work advances these benchmarking efforts by incorporating a biological case study - IL-6 signalling in myeloma - showing how these protocol-specific technical biases directly propagate into differential expression, pathway enrichment, and isoform usage analyses.

Pragmatically, protocol choice should be guided by study aims. For isoform discovery, differential transcript usage, and fusion detection, dRNA sequencing offers higher confidence despite greater RNA input and low throughput. For experiments prioritising sensitivity to low-abundance transcripts or when input is limiting, PCR-cDNA remains valuable. Taken together with prior benchmarking efforts, our analysis provides a technical framework for selecting ONT sequencing protocols and for interpreting their results with appropriate caution, particularly when drawing biological conclusions from isoform-level data.

## Methods

### SKMM2 cell culture and RNA extraction

SKMM2 cells (DSMZ: ACC 430) were cultured in Gibco™ RPMI 1640 medium (11530586, Fisher Scientific, UK), supplemented with 10% FBS (SV30160.03, Cytiva Life Sciences, UK), and maintained at 37 °C in a 5% CO₂ atmosphere. Cells were seeded into 12-well plates in triplicate and treated with either 1 ng/mL interleukin-6 (IL-6) in 0.2% BSA or with 0.2% BSA alone for 18 h. Following treatment, cells were harvested and cell pellets resuspended in 350 µL Trizol reagent. RNA extraction was performed using the Zymo Direct-zol RNA Miniprep kit (R2051, Zymo Research, USA) according to the manufacturer’s protocol, with RNA eluted in 25 µL DNase/RNase-free water. RNA concentration was determined using a NanoPhotometer NP80 UV/Vis Spectrophotometer (Implen, Germany), while RNA integrity and size profile were assessed using the Agilent 4150 TapeStation System (Agilent Technologies, UK) with an RNA ScreenTape (Agilent Technologies, UK). All RNA samples had a RNA Integrity Number (RIN) between 9.7 and 9.8 (Supplementary Fig. 3 and Supplementary Fig. 4).

### PCR-cDNA library preparation and sequencing

Library preparation was performed using the PCR-cDNA Barcoding Kit (SQK-PCB114.24, Oxford Nanopore Technologies, UK) following the manufacturer’s instructions. A total of 500 ng of RNA (IL-6 treated, *n* = 3; untreated, *n* = 3) or 16 ng of ERCC RNA Spike-In Mix 1 (Thermo Fisher, UK) was used for cDNA synthesis. For SKMM2 cell line samples, 14 cycles of PCR amplification were conducted, while 16 cycles were used for the ERCC mix (Supplementary Fig. 5). Library concentrations were quantified using a Qubit 2.0 Fluorometer (Thermo Fisher, UK) with the Qubit dsDNA HS Assay kit (Q32851, Thermo Fisher, UK). Library quality was assessed on a 4150 TapeStation System (Agilent Technologies, UK) using a high sensitivity D5000 ScreenTape. For sequencing, 5 fmol of each barcoded library were pooled, and 6 libraries (30 fmol) were loaded onto a PromethION R10.4.1 flow cell (FLO-PRO114M, Oxford Nanopore Technologies, UK). Sequencing was performed on a PromethION 2 Solo instrument (Oxford Nanopore Technologies, UK) using MinKNOW software (v25.05.14), with live base-calling using the high-accuracy model 400bps for 53 h. The Dorado software version integrated into MinKNOW was v7.9.8. For the ERCC mix, 5 fmol of the prepared library was loaded onto an R10.4.2 Flongle flow cell (FLO-FLG114, Oxford Nanopore Technologies, UK), and sequencing was conducted on a MinION Mk1B instrument with the Flongle adapter (Oxford Nanopore Technologies, UK). The MinKNOW software (v24.02.16) was used to control the run with live base-calling using the High-accuracy model 400bps, with a 24-hour run time and a minimum read length of 20 bp. Live basecalling using high accuracy was performed using the Dorado basecaller integrated within MinKNOW (MinKNOW v25.05.14; embedded Dorado v7.9.8). In both sequencing runs reads with a minimum Q score of 9, MinKNOW’s default setting at the time, were classed as ‘passed’ to filter out low-quality reads.

### Direct RNA library preparation and sequencing

Library preparation was performed using the Direct RNA Sequencing Kit (SQK-RNA004, Oxford Nanopore Technologies, UK) following the manufacturer’s instructions. A total of 1 µg of RNA (IL-6 treated, *n* = 3; untreated, *n* = 3) or ~ 150 ng of ERCC RNA Spike-In Mix 1 (*n* = 1) was used for dRNA sequencing. The concentration of the prepared libraries was measured using a Qubit 2.0 Fluorometer (Thermo Fisher, UK) and the Qubit dsDNA HS Assay kit (Q32851, Thermo Fisher, UK). All libraries exceeded the recommended 30 ng yield. Library size profiles were assessed using the Agilent 4150 TapeStation System (Agilent Technologies, UK) with a high sensitivity D5000 ScreenTape. Libraries were loaded in their entirety onto separate PromethION RNA Flow Cells (FLO-PRO004RA, Oxford Nanopore Technologies, UK). Sequencing was performed on a PromethION 24 instrument (Oxford Nanopore Technologies, UK) with MinKNOW software (v24.06.14). Live basecalling using high accuracy was performed using the Dorado basecaller integrated within MinKNOW (MinKNOW v25.05.14; embedded Dorado v7.9.8). Sequencing lasted for 72 h, reads with a minimum read length of 200 bp and a minimum Q score of 9 were classed as ‘passed’ to filter out low-quality reads, as per MinKNOW’s default settings at the time.

### Data pre-processing

Reads passing quality filters were demultiplexed by MinKNOW software and concatenated into a single FASTQ file for each sample. For PCR-cDNA data, adapter trimming and read orientation were performed using pychopper (v2.7.10, Oxford Nanopore Technologies, UK). For SKMM2 PCR-cDNA libraries, reads shorter than 200 bp were discarded, for the ERCC PCR-cDNA library this was set to 50 bp. To control for potential depth-related biases SKMM2 PCR-cDNA and dRNA reads were randomly subsampled to 4 million reads per sample using Seqtk, random seed 200.

### Alignment

All reads were aligned to the hg38 human genome assembly (GRCh38.p14, GENCODE v46) using minimap2 (v2.28) with spliced alignment mode, a k-mer size of 14, a minimum primary-to-secondary score ratio of 0.9 and canonical splice-site searching on the forward strand only.

### Transcript abundance estimation

Gene and transcript count matrices for SKMM2 cell line samples were generated using bambu (v3.8.3) in R (v4.4.0). Transcript quantification of ERCC reference transcripts was performed using featureCounts within the Subread package (v2.0.6).

Transcript-level confidence of SKMM2 cell line samples were assigned based on total read support across all samples. Transcripts were classified as high-confidence if they had > 5 total reads in both unique and full-length counts. Low-confidence transcripts were classified if they had zero reads in either unique and full-length counts. Remaining unclassified transcripts were classed as medium-confidence.

### Read characteristics and transcript coverage

Read lengths were calculated using the fastq read lengths module of BioKit (v0.5.0). Read statistics were generated using long-read tool Nanoplot (v1.46.2). Percentile-wise (5’-3’) coverage of the CDS of all known transcripts was calculated using the genebodycoverage module of the RSeQC package (v5.0.4) on a 4 million read subsample from each BAM file. The analysis was then repeated including only transcripts > 1000 bp in length. Percentile-wise coverage values were normalized against the maximum value per sample (i.e. the percentile with the highest coverage) and then the mean of each protocol was calculated. The proportion of full-length transcripts was calculated as a percentage of total unique transcript counts assigned by bambu (v3.8.3).

### Differential gene expression

Differential gene expression was analysed using DESeq2 (v1.44.0) with a false discovery rate (FDR) of 5%. Count normalization was performed using variance stabilizing transformation (VST), and VST-normalized data were used for visualisation and exploratory analyses. Gene Set Variation Analysis (GSVA) was performed using the GSVA package (v2.0.7) with hallmark gene sets from the Molecular Signatures Database (MSigDB). Differential expression of gene sets was assessed using linear modelling with empirical Bayes moderation (FDR = 5%).

### Differential transcript usage

Prior to testing for differential transcript usage (DTU), genes and transcripts were filtered using DRIMSeq (v1.52.0) [20]. Genes were required to be expressed in all samples at > 5 counts, and transcripts had to be expressed in > 3 samples, with > 5 counts and > 10% transcript usage for the gene. DTU analysis was conducted using DEXSeq (v1.52.0), with FDR set to 5% for both gene-wise and transcript-wise analyses. Stage-wise adjustment for DTU was performed using StageR (v1.28.0) with FDR set to 5%, as described by van den Berge et al. Graphics were produced using ggplot2 (v4.0.1), ggpubr (v0.6.2), ggvenn (v0.1.19), ggtranscript (v1.0.0), ggalluvial (v0.12.5), and pheatmap (v1.0.13).

### Novel transcript discovery

Novel transcript discovery was performed using bambu (v3.1) in R, combining all 12 samples and applying a novel discovery rate threshold of 0.1. Novel transcripts were considered expressed if supported by ≥ 5 in at least two biological replicates. Genes annotated as “novel genes” that were < 300 bp or overlapped annotated gene loci were manually excluded as likely artefacts. Differential transcript expression (DTE) analysis was performed using DESeq2 (v1.46.0) with contrasts defined as IL6 vs. WT and cDNA vs. dRNA. Prior to analysis, transcripts were pre-filtered to retain those with ≥ 5 counts in at least three samples. Results were filtered to retain significantly differentially expressed transcripts (padj < 0.05), and downstream visualization (volcano plots and heatmaps) was restricted to novel transcripts to compare differential expression between treatment conditions and sequencing protocols.

### Fusion gene detection

Gene fusions were identified using five independent pipelines (JAFFAL, CTAT-LR, FusionSeeker, LongGF, and FLAIR-fusion), with breakpoint re-annotation to identify consensus fusion pairs across the pipelines, prior to filtering; fusion events were retained if detected by ≥ 2 callers with ≥ 3 supporting reads and with partner loci separated by ≥ 300 kb.

### Immunoglobulin enrichment analysis

Immunoglobulin (IG) transcripts were identified from the Ensembl v46 annotation as those whose HGNC symbol began with IGH, IGK or IGL, and were assigned to a segment class (V, D, J or C) based on their Ensembl biotype. Gene-level read counts were averaged across the six replicates of each protocol, and a gene was considered expressed when its higher protocol mean was ≥ 100 reads. Read-length distributions were computed separately for the pooled PCR-cDNA and dRNA reads, binned at 10 bp resolution and expressed as the proportion of reads per bin. Within each distribution the dominant peak was identified as a local maximum and its boundaries were defined as the full width at half maximum (FWHM), giving peaks of 384–602 bp (mode 535 bp) for PCR-cDNA and 905–946 bp (mode 925 bp) for dRNA. The IGK read fraction was calculated per sample as the number of reads assigned to IGK genes divided by the total assigned gene-level reads, and summarised as the mean across the six replicates of each protocol.

## Supplementary Information

Supplementary Material 1.

Supplementary Material 2.

Supplementary Material 3.

## Data Availability

Sequencing data have been deposited to the Gene Expression Omnibus under the accession number GSE306225. All analysis was performed using hg38 ensembl 101 version.

## Code availability

All custom code used within this analysis are available on GitHub (https://github.com/cribbslab/ONT_comparison).
