# Supplementary material for: Distinct 5′ and 3′ coverage biases shape transcriptome interpretation in Nanopore direct RNA versus PCR-cDNA sequencing
Source: BMC Genomics. 2026 Jul 15;27:827. doi: 10.1186/s12864-026-13184-x (PMC13647511; doi:10.1186/s12864-026-13184-x)
Supplement: Supplementary file 1 [file 12864_2026_13184_MOESM3_ESM.docx]

**Supplementary Figure 1. Immunoglobulin (IG) transcripts drive the characteristic read-length peaks in SKMM2 cells.**

(a) Mean expression counts of top IG genes in SKMM2 cells show an enrichment pattern characteristic of plasma cell malignancies. Bars show log10(mean count + 1) for control and IL6-treated cells (N=6, per protocol). (b) Annotated transcript isoform lengths of IG segment genes. Each dot represents one annotated transcript isoform. Dot size is proportional to the mean expression of the parent gene (log10(max mean count +1), where max mean count is the larger of the mean PCR-cDNA or dRNA count. Dot colour represents the IG segment class. Faded dots represent transcripts below the expression cutoff (max mean counts < 100) in SKMM2 cells. Grey reference bands denote observed read-length distribution peaks per protocol. (c) Schematic of the rearranged SKMM2 clonal IGK light chain transcript. IGK genes are highlighted within the expected rearrangement of the IGK transcript with IGKV1-6, IGKJ1, and IGKC giving a total predicted length of ~950bp, consistent with the peak observed in the dRNA dataset. PCR-cDNA reads are more enriched for the IGKV1 (~350-410bp) and IGKC (523bp) region gene segments suggesting a limited capture of full-length transcripts by the PCR-cDNA dataset compared to the dRNA dataset. (d) Read-length distribution (10 bp bins) of all reads per protocol. Grey bands mark each peak ± its half-maximum width (PCR-cDNA 384–602 bp, mode 535 bp; dRNA 905–946 bp, mode 925 bp); dotted lines mark IGK reference lengths.

**Supplementary Figure 2: IGK fraction of assigned reads in SKMM2 cells.** Percentage of total assigned reads attributable to IGK genes for each sequencing protocol, from a mean of 3.4 million (PCR-cDNA) and 3.8 million (dRNA) assigned reads per sample. Bars show the mean (2.8% for PCR-cDNA, 5.7% for dRNA) and open circles show individual samples (N = 6 per protocol). IGK is the single most highly expressed gene family in SKMM2, consistent with the defining kappa light-chain secretion of this cell line (Eton et al., 1989), and is more enriched in the dRNA libraries.

**Supplementary Figure 3:** Proportion of full-length transcripts captured by PCR-cDNA and dRNA sequencing of all SKMM2 cell line samples (control and IL6-treated). Overall, PCR-cDNA libraries exhibited a higher proportion (42.9%) of full-length transcripts compared to dRNA libraries (33.4%). Black dots represent the percentage of full-length transcripts per sample (N=6, per protocol).

**Supplementary Figure 4:** Quality control of RNA with TapeStation System's High Sensitivity RNA ScreenTape.

The electropherogram profiles show the size distribution of the ladder (A), Control replicate 1 (B), Control replicate 2 (C), Control replicate 3 (D), IL6 replicate 1 (E), IL6 replicate 2 (F), and IL6 replicate 3 (G). All libraries had a RINe of >9.4, showing little RNA degradation.

**Supplementary Figure 5:** Quality control of PCR-cDNA libraries with TapeStation System's High Sensitivity D5000 ScreenTape.

The electropherogram profiles show the size distribution of the ladder (A), Control replicate 1 (B), Control replicate 2 (C), Control replicate 3 (D), IL6 replicate 1 (E), IL6 replicate 2 (F), and IL6 replicate 3 (G). The reported concentrations of the PCR-cDNA libraries were between 0.272ng/ul - 0.905ng/ul with an average size of between 926-1250bp.

**Supplementary Figure 6:** Quality control of PCR-cDNA ERCC synthetic spike-ins library with TapeStation System's High Sensitivity D5000 ScreenTape. The electropherogram profiles show the size distribution of the ladder (A) and the PCR-cDNA library made from ERCC synthetic spike-ins (B). The reported concentration of the ERCC PCR-cDNA library was 0.629ng/ul with an average size of 957bp and noticeable peaks at 145bp, 313bp, 416bp, 669bp, 1200bp, and a less abundant peak at 2110bp.
